# Continental‐scale empirical evidence for relationships between fire response strategies and fire frequency

**DOI:** 10.1111/nph.20464

**Published:** 2025-02-11

**Authors:** Sophie Yang, Mark K. J. Ooi, Daniel S. Falster, William K. Cornwell

**Affiliations:** ^1^ Evolution & Ecology Research Centre, School of Biological, Earth & Environmental Sciences University of New South Wales Sydney NSW 2052 Australia; ^2^ Centre for Ecosystem Science, School of Biological, Earth & Environmental Sciences University of New South Wales Sydney NSW 2052 Australia

**Keywords:** Australia, fire frequency, fire intensity/severity, leaf traits, life history, resprouting, seeding

## Abstract

- Theory suggests that the dominance of resprouting and seeding, two key mechanisms through which plants persist with recurrent fire, both depend on other traits and vary with fire regime. However, these patterns remain largely untested over broad scales.
- We analysed the relationships between mean fire frequency, derived from MODIS satellite data, and resprouting and seeding strategies, respectively, for *c*. 10 000 woody and herbaceous species in Australia. We tested whether leaf economics traits differed among these strategies.
- Probability of resprouting exhibits a monotonic increase with fire frequency for woody plants; for herbaceous plants, a hump‐shaped relationship is observed. Probability of seeding exhibits a hump shape with fire frequency in woody plants. In herbaceous plants, probability of resprouting was associated with higher leaf mass per area (LMA), and probability of seeding with lower LMA. A broader range of leaf investment strategies occurred in woody plants.
- Our findings provide the largest empirical support to date for theory connecting fire response strategy to fire frequency. Woody seeders appear constrained by immaturity and senescence risk. Herbaceous and woody seeders showed different placements along the leaf economics spectrum, suggesting an important interaction between growth form and growth rate for seeders.

## Introduction

Fire is a fundamental ecological process for many ecosystems on Earth (Bond & Keeley, 2005; Andela *et al*., 2019; McLauchlan *et al*., 2020), which shapes the evolution of traits that allow organisms to thrive in fire‐prone environments (Keeley *et al*., 2011; He & Lamont, 2018; Pausas & Bond, 2019). A key focus of research has been the mechanisms that enable plants to persist in fire‐prone environments. Past research has identified two main strategies adopted by plant species: resprouting from surviving tissues (hereafter ‘resprouting’) and postfire germination from seed (hereafter ‘postfire seeding’) (Lamont *et al*., 1991; Whelan, 1995; Bond & van Wilgen, 1996). While some species are capable of one mechanism alone, so‐called ‘obligate’ resprouters and seeders, others are capable of both, called ‘facultative’ species. Theoretical and empirical research surrounding these mechanisms suggests that the two strategies exist at opposite ends of a spectrum of resource allocation (Iwasa & Kubo, 1997; Bell, 2001; Bowen & Pate, 2017), with varying costs and benefits, and depending on the fire regime. It follows that patterns in the relative proportion of these fire response strategies could vary across gradients of fire regime characteristics, such as fire frequency or severity. Although established in theory (Hilbert, 1987; Bellingham & Sparrow, 2000; Bond & Midgley, 2003; Pausas & Keeley, 2014b), these patterns have remained largely untested on a broad scale, mainly due to the lack of large‐scale data on fire regimes and species regeneration mechanisms.

In fire‐prone ecosystems, the success of different regeneration mechanisms is dependent on the fire regime. A fire regime captures the typical event‐ and frequency‐driven characteristics of fires in a given place within an ecological time frame (Gill, 1975) and comprises multiple parameters, including fire frequency, intensity (rate of heat energy release), severity (biological impacts on above‐ and below‐ground vegetation), type (ground, surface, crown, mixed), size and seasonality (McLauchlan *et al*., 2020). Fire regimes vary markedly between vegetation types, such as in the mesic forests of eastern Australia and boreal forests of North America where fires burn infrequently but often intensely (Gill & Catling, 2002; Keeley & Pausas, 2022), vs the tropical and subtropical savannas in southern Africa and South America which undergo frequent, low‐intensity fires (Archibald *et al*., 2013; Lehmann *et al*., 2014). Two major drivers of selection on plant response strategies are fire frequency and severity. Fire frequency defines the length of time between fires in which plants can grow and maintain resprouting organs and/or reach reproductive maturity and produce a sufficient seed bank. Severity relates to the amount of biomass consumed and levels of plant mortality and reproductive success. Severity is influenced by the intensity of fire, which is in turn dependent on frequency, with frequent fire regimes constrained to mostly moderate‐intensity fires and rare fire regimes capable of producing both low‐ and high‐intensity fires (Archibald *et al*., 2013). Here, we focus on one major fire regime characteristic, fire frequency, which has been at the core of theory relating to plant–trait relationships (Hilbert, 1987; Bellingham & Sparrow, 2000; Bond & Midgley, 2003; Pausas *et al*., 2004). However, the interplay of fire frequency and severity necessitates their joint interpretation. Globally, ecosystems range from experiencing frequent fire, close to 100 fires per century, to very infrequent fire, less than one fire per century (Archibald *et al.*, 2013).

Theory suggests that the frequency of resprouting and postfire seeding strategies of woody plants should vary with factors that modify the relative survivorship of adult and juvenile plants (Pausas & Keeley, 2014b). Pausas & Keeley (2014a) likened obligate resprouting and obligate seeding strategies to perennial and annual life history strategies, when fire events are considered equivalent to annual cycles. Obligate resprouters follow a longer‐lived, ‘perennial’ life history as they live and reproduce through many fire intervals (iteroparity) (Pausas & Keeley, 2014a). Conversely, obligate seeding species follow a shorter‐lived, ‘annual’ life history, with mature individuals suffering high mortality after severe fire and thus depending on a single reproductive event per generation (semelparity) (Bond & van Wilgen, 1996; Pausas & Keeley, 2014a). Pausas & Keeley (2014a) proposed that selection towards these opposing strategies depends on the ratio of parent to offspring survivorship; resprouting should be selected for when adult plants are more likely to survive a fire relative to juveniles. Resprouting is therefore expected to be more common under less severe fire regimes, and varying levels of fire severity determine the effectiveness of different types of resprouting (basal, epicormic and axillary bud) (Moreira *et al*., 2009; Kenefick *et al*., 2024). Facultative species exist between these extremes, resprouting and seeding being nonmutually exclusive strategies.

Along with fire severity, fire frequency is also a major driver of selection on resprouting and seeding strategies. Investment in resprouting capacity may be wasted when fires are very rare, but may also be inviable in environments where fires are both frequent and severe, as there is insufficient opportunity for recovery between disturbances (Iwasa & Kubo, 1997; Bellingham & Sparrow, 2000; Pausas & Keeley, 2014a). Bellingham & Sparrow (2000) hypothesised that the ability to resprout would increase as the frequency of severe disturbances increases, until a threshold where resprouting becomes less viable and so ability to resprout then declines. Similarly, postfire seeding is predicted to be more prevalent at intermediate fire frequencies (Hilbert, 1987; Lamont *et al*., 1991), as a balance between two competing risks. Seeders suffer an immaturity risk when fire return intervals (FRIs) are too short (Keeley *et al*., 1999), but can also experience a senescence risk when FRIs are longer than the longevity of the plants and seed bank combined (Keeley, 1986).

While discussion around plant–fire relationships has predominantly been centred around woody plants, herbaceous plants have been comparatively understudied, despite being an important group that makes up much of the world's most fire‐prone ecosystems (Mouillot & Field, 2005; Keeley & Pausas, 2022). We define ‘woody’ as having a prominent aerial stem that lasts through time and changing environmental conditions (Zanne *et al*., 2014); in practice, this corresponds to a longer lifespan of the aboveground part of the plant as well as a taller potential height. Recently, Simpson *et al*. (2021) extended the model by Bellingham & Sparrow (2000) to grasses; however, a small difference in their predictions for grasses was that, although resprouting may decline at very high fire frequencies, it could still be a common strategy, as many grasses can resprout from protected underground buds or insulated leaf bases (Klimešová & Klimeš, 2003; Simpson *et al*., 2021). Additionally, herbaceous seeders were hypothesised to be less restricted by immaturity risk as their time to maturation is relatively short, supported by findings that the ratio of grass seeders to resprouters was higher at very high frequencies (Simpson *et al*., 2021).

To fulfill contrasting life histories, seeders and resprouters may also employ different strategies of resource allocation and growth. Seeders might allocate more resources to rapid aboveground growth and early reproduction, whereas resprouters might allocate more resources to storage organs and protective structures that improve survival and regrowth after fire (Iwasa & Kubo, 1997; Bell, 2001; Pausas *et al*., 2004; Bowen & Pate, 2017). These resource allocation strategies could also be manifested in leaf economics traits (Wright *et al*., 2004), such as leaf mass per area (LMA) and leaf nitrogen (N) content. Fast‐growing seeders are expected to have ‘quick‐return’ leaves, with low LMA, high leaf nutrients (including N), high leaf turnover and high rates of photosynthesis and respiration, whereas the opposite is expected for slower‐growing resprouters (Wright *et al*., 2004). Past studies investigating leaf traits across fire response strategies have found varying results across climates and ecosystems (Ackerly, 2004; Pausas *et al*., 2004; Paula & Pausas, 2006; Saura‐Mas & Lloret, 2007; Vivian & Cary, 2012), thus raising questions about how generally leaf traits relate to fire strategy. Moreover, the fundamental differences between woody and herbaceous growth forms may lead to different patterns of leaf traits across fire response strategies (e.g. in grasses; Simpson *et al*., 2021). Generally, it is known that herbaceous and woody species differ in their leaf traits (Towers *et al*., 2024), but it remains unclear whether there are further differences within herbaceous species with respect to fire response, and whether such differences are consistent across herbaceous and woody species. In addition to fire history, both leaf traits and the proportion of resprouters relate to plant lifespan, with very short lifespan species (i.e. annuals) thought typically not to resprout following fire. Thus, nonfire climatic factors that ecologically select for very short lifespan species may also lead to a greater proportion of nonresprouters. Whatever its origin, the inability of species in these climates to resprout following fire may still have important implications as fire moves into both new seasons and new parts of the world.

Despite an abundance of theory, the consequences of fire have rarely been evaluated at large biogeographic scales, with data to span hundreds to thousands of species. However, a flourishing of biodiversity resources, such as species distribution, fire occurrence and trait data, now enables such questions to be addressed. Recently, Simpson *et al*. (2021) tested the effects of fire frequency on the distribution of 734 grass species, globally. While this global analysis of grasses represented a significant advance in our understanding of plant–fire relationships for herbaceous species, the scope of the study was still constrained to just a single plant family with a particular adaptation: having a basal meristem. Here, we use large‐scale empirical data to investigate the distribution of fire response strategies at an unprecedented scale, by quantifying characteristics for > 9500 species spread across the entire continent of Australia. Specifically, we ask the following questions:

1. What is the distribution of fire frequencies experienced by Australian plant taxa?
2. Do the fractions of plant species that are resprouting and seeding support the hypothesised hump‐shaped response with fire frequency, in both woody and herbaceous plants?
3. Within woody and herbaceous species, do leaf traits (LMA and leaf N content) differ between resprouters and nonresprouters, or between postfire seeders and nonseeders?

## Materials and Methods

### Study system

Our study area is the continent of Australia, which contains deserts, savanna, tropical and subtropical forests, temperate and Mediterranean woodlands and shrublands, spread across large climatic gradients (Keith, 2017). This range of ecosystems and component species representing different life histories and fire response strategies as well as a large range of fire frequencies (Bradstock, 2010; Murphy *et al*., 2013) is representative of those observed globally, and thus provides an ideal region to empirically test the theory connecting resprouting and seeding to fire frequency (Bellingham & Sparrow, 2000; Pausas & Keeley, 2014b).

### Fire response and leaf traits

We extracted fire response trait data for Australian plants from AusTraits (v.5.0.0), a database that includes data on the fire response strategies of *c*. 9578 species (over 40% of all described Australian vascular plants; CHAH, 2023) (Falster *et al*., 2021). The database harmonises trait data from diverse sources, including field studies, herbaria and published literature. Our two traits of interest, whether a taxon resprouts and whether it has postfire seeding, are captured by ‘resprouting_capacity’ and ‘post_fire_recruitment’ (Wenk *et al*., 2024c). The resprouting trait (‘resprouting_capacity’) distinguishes whether individuals in a species or population are killed by fire (< 30% resprout), partially resprout (30–70%) or resprout prolifically (> 70%). The postfire seeding trait (‘post_fire_recruitment’) specifies whether a plant does or does not display increased germination postfire. We considered resprouting and postfire seeding as separate traits given that the traditional resprouter/seeder dichotomy does not capture species that can do both (1672 species or 15% of our dataset was facultative).

Before categorising species as having capacity for postfire resprouting and postfire seeding, we filtered out non‐native species by only including those that are native in at least one state or territory according to data from the Australian Plant Census (APC) (CHAH, 2023). We excluded hybrid species and species names that were not currently accepted by the APC. We included fire response trait observations at the infraspecific level (form, variety and subspecies) in addition to the species level, because within‐species differences in fire response strategies, or similarities in strategies despite exposure to different fire regimes, are also informative. We also ran analyses without infraspecific taxa to determine potential over‐counting, since they are likely to share traits with their species‐level counterparts. We determined the number of infraspecific taxa that had different trait values to the species‐level value. Infraspecific and species‐level taxa (terminal taxa) are henceforth collectively referred to as ‘species’. Each species was then categorised as a resprouter or nonresprouter and a (postfire) seeder or nonseeder. In AusTraits, observations from multiple sources can be recorded for a species in any given trait. Hence, we classified a species as a resprouter if at least 30% of observations recorded resprouting or partial resprouting, and a seeder if at least 30% of observations recorded postfire recruitment. The absence of seedlings is more difficult to detect than their presence, possibly leading to an inclusion bias towards seeders. Lastly, species were identified as woody or herbaceous using ‘woodiness_detailed’ data from the curated Australian plant growth forms dataset, ‘Wenk_2022’ (Wenk *et al*., 2024a). We categorised records that were combinations of trait values, for example ‘herbaceous semi_woody’, into groups: woody, herbaceous, semi‐woody and ambiguous (Supporting Information Table S1), the last two of which we discarded. This resulted in a dataset of 6387 woody species and 4634 herbaceous species. 10 881 species (6276 woody, 4605 herbaceous) had data on resprouting and 3570 species (2661 woody, 909 herbaceous) had data on postfire seeding.

We also extracted data on two leaf traits, LMA and leaf N content (per unit dry mass), and life history from the AusTraits database, filtering out non‐native species. In AusTraits, each observation is on a separate row and values for a numerical trait are standardised to the same units. The value type, for example raw value or mean, and the number of replicates used to calculate each observation (if a mean) are also recorded in separate columns. To obtain species‐level measures of LMA and leaf N, we took the mean of all LMA and leaf N observations, respectively, for the species. The mean was calculated by dividing by the number of rows (observations), rather than by the number of replicates used for each observation, because the majority of observations were raw values (single replicates) and the numbers of replicates used to calculate means were often unreported. 2844 species (2061 woody, 783 herbaceous) had data on LMA and 1637 species (1385 woody and 252 herbaceous) had data on leaf N, in addition to data on at least resprouting or seeding. Life history was extracted from a curated dataset, ‘Wenk_2023’, which contains data from state and national online floras (Wenk *et al*., 2024a). Where a species was scored as both ‘annual’ and ‘perennial’ (454 species), we assigned ‘perennial’, since these have the potential to be perennial and we were interested in whether ‘obligate’ annuals would influence leaf traits and proportions of resprouters/seeders.

### Species occurrences

We extracted species occurrences from the Global Biodiversity Information Facility (GBIF). On the GBIF web portal, we selected ‘present’ occurrence status records of vascular plants located in Australia (GBIF.org (2 June 2023) GBIF Occurrence Download DOI: 10.15468/dl.phfr9u). GBIF records were subsequently cleaned in the R environment (R Core Team, 2021). Occurrence records were filtered to post‐1900, georeferenced human observation and living and preserved specimen records only. Coordinates with low precision (< 2 decimal digits) and high uncertainty (> 10 000 m) were excluded, as well as coordinates with longitude or latitude recorded as zero. We removed duplicate records and records flagged with the ‘country coordinate mismatch’ and ‘recorded date unlikely’ issues in GBIF. A series of checks were conducted with the {coordinatecleaner} package (v.2.0‐20; Zizka *et al*., 2019), including removing nonterrestrial and unlikely or invalid coordinates. Coordinates in the ocean or within 2 km of the country centroid, capital centroid and biological institutions were also removed. 13 605 512 observations remained from 21 141 315 after filtering (36% of observations were removed). Species names from GBIF, which use the World Checklist of Vascular Plants (WCVP) backbone, were aligned with names in AusTraits, which are standardised with the APC. 10 871 species were matched between AusTraits and GBIF after aligning and updating GBIF names using the {apcalign} package (Wenk *et al*., 2024b).

### Fire frequency

We aimed to determine the mean fire frequency that each species is exposed to across its distribution. Data on fire events were extracted from the Moderate Resolution Imaging Spectroradiometer (MODIS) Global Monthly Burnt Area Data Product (MCD64A1), which contains the spatial extent and dates of fires occurring from November 2000 to October 2022 at a 500‐m resolution. The MODIS satellite detects burn scars by identifying daily changes in surface reflectance after a fire (e.g. vegetation loss and charcoal deposits), which is supplemented with daily active fire data (Giglio *et al*., 2018). Although only a *c*. 22‐yr dataset, MODIS data are the best available fire data for Australia at the continental scale.

**Fig. 1:**
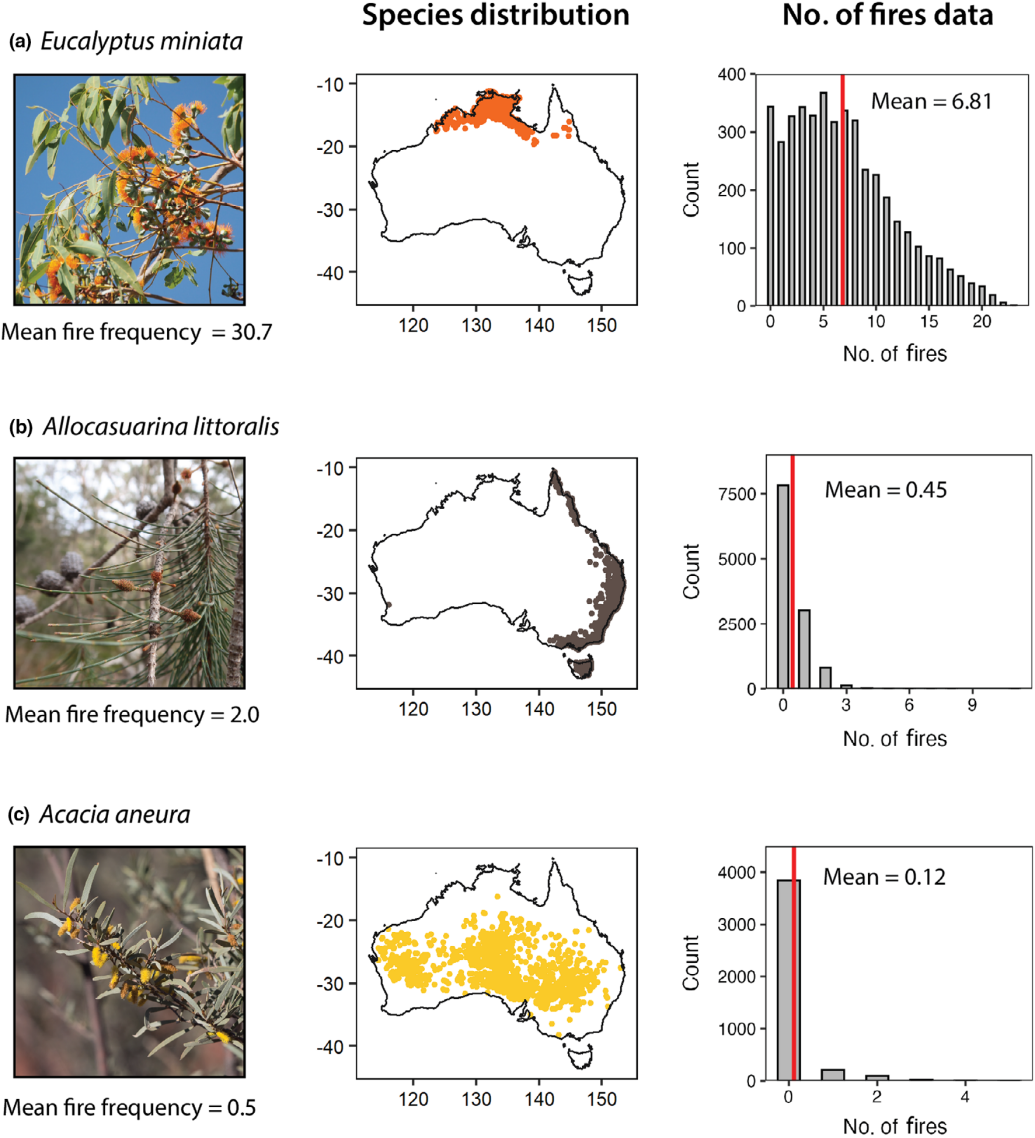
Schematic illustrating mean fire frequency (per century) for three example species. Centre panels show the known distribution, as represented by observations from the Global Biodiversity Information Facility. The right panels show the number of fires data in each 500 × 500 m cell during the period 2000–2022, across each species' range, with mean event rate across the observation period signified by the red lines. Creators of plant images are discoverable on iNaturalist: (a) *Eucalyptus miniata* (Jenny Donald (CC‐BY‐NC)), (b) *Allocasuarina littoralis* (@cowirrie (CC0)) and (c) *Acacia aneura* (Kym Nicolson (CC‐BY)).

First, we extracted the occurrence records of a given species and overlaid this with the MODIS data product (500‐m grid cells). In each grid cell where the species occurs, we counted the number of fires that had occurred during the *c*. 22‐yr period. We removed species with < 10 total cells or entirely unburnt cells (622 species). We then calculated the mean event rate across the observation period, which was the mean number of fires the species had experienced across its cells. Mean event rates were converted to mean fire frequency per century (Fig. S1). Examples of species' spatial distributions and their fire event count data are displayed in Fig. 1.

Using the mean of fire count data assumes a constant rate of fire events occurring through time, which is unlikely true for many systems, because of factors like increasing fire risk with accumulating fuel loads and decreasing fire risk from negative fire–vegetation feedbacks (Fernandes *et al*., 2012; Héon *et al*., 2014). However, the true rate of fire over time is highly debated in fire literature (Moritz, 2003; Moritz *et al*., 2004; Keeley & Zedler, 2009), and hence, we used a constant rate as a compromise between increasing and decreasing rates. Also, accepting a different assumption of increasing or decreasing rate of fire would only change the absolute values of fire frequency estimates and have no effect on the absolute ranking of fire frequencies across species, which was sufficient for our analysis. To check our method was producing reasonable numbers, we also compared values to those estimated using a method by Simpson *et al*. (2021), which calculates the median FRI from fitting a Weibull distribution to all the inter‐fire intervals across the grid cells in which a species occurs (Fig. S1). The distribution of fire frequencies across the two methods was largely similar, with the survival analysis method more skewed towards low fire frequencies (Fig. S2).

### Data analysis

We analysed the relationships between fire response strategies and fire frequency for woody and herbaceous species using a generalised linear model with a logit link and binomial response in R (R Core Team, 2021). Two separate models were fit with resprouting and postfire seeding as the response variables and fire frequency (per century) as the predictor variable. We fit a quadratic polynomial function, based on *a priori* hypotheses formed from the literature (quadratic curves; Bellingham & Sparrow, 2000; Simpson *et al*., 2021) and inspection of the raw data. Resprouting and postfire seeding were coded as binomial (i.e. TRUE or FALSE equates to resprouts or does not resprout), while fire frequency was log‐transformed to reduce skewness and leverage of low fire frequencies. We included a woody or herbaceous variable and any interactions with fire frequency in the resprouting model. For the postfire seeding model, however, we only included woody species as there were insufficient data to fit the model for herbs (909 herbaceous species vs 2661 woody species). To determine whether results would be influenced by life history, we removed annuals (690 species) and species missing life history data (33 species) and repeated the analyses. Additionally, to determine whether accounting for evolutionary relationships would affect the results, we repeated the analyses with phylogenetic logistic regression (Ives & Garland, 2010) using the phyloglm function (method set to ‘logistic_MPLE’ or maximised penalised likelihood of the logistic regression) from package {phylolm} (Ho & Ané, 2014). We used a dated phylogeny for seed plants by Smith & Brown (2018), which contains 356 305 species and combines genetic data from GenBank and phylogenetic data from the Open Tree of Life project. The tree was subset to the species in our study; however, due to differences in taxonomy, 2587 species were unable to be matched (25.5% of data).

To determine how leaf traits compare between fire response strategies, we fit four linear regressions with LMA and leaf N as individual response variables (log‐transformed) and resprouting or postfire seeding as explanatory variables (R Core Team, 2021). We included a woody or herb variable and an interaction term as additional explanatory variables in each model. We repeated the analyses without annuals, and also with phylogenetic linear regression using the same phylogenetic tree as previously (Smith & Brown, 2018) and the phylolm function from package {phylolm} (Ho & Ané, 2014). *P‐*values of estimated marginal means are adjusted with the Tukey method for standard linear regression and with the Bonferroni method for phylogenetic linear regression.

## Results

### Fire frequency

**Fig. 2:**
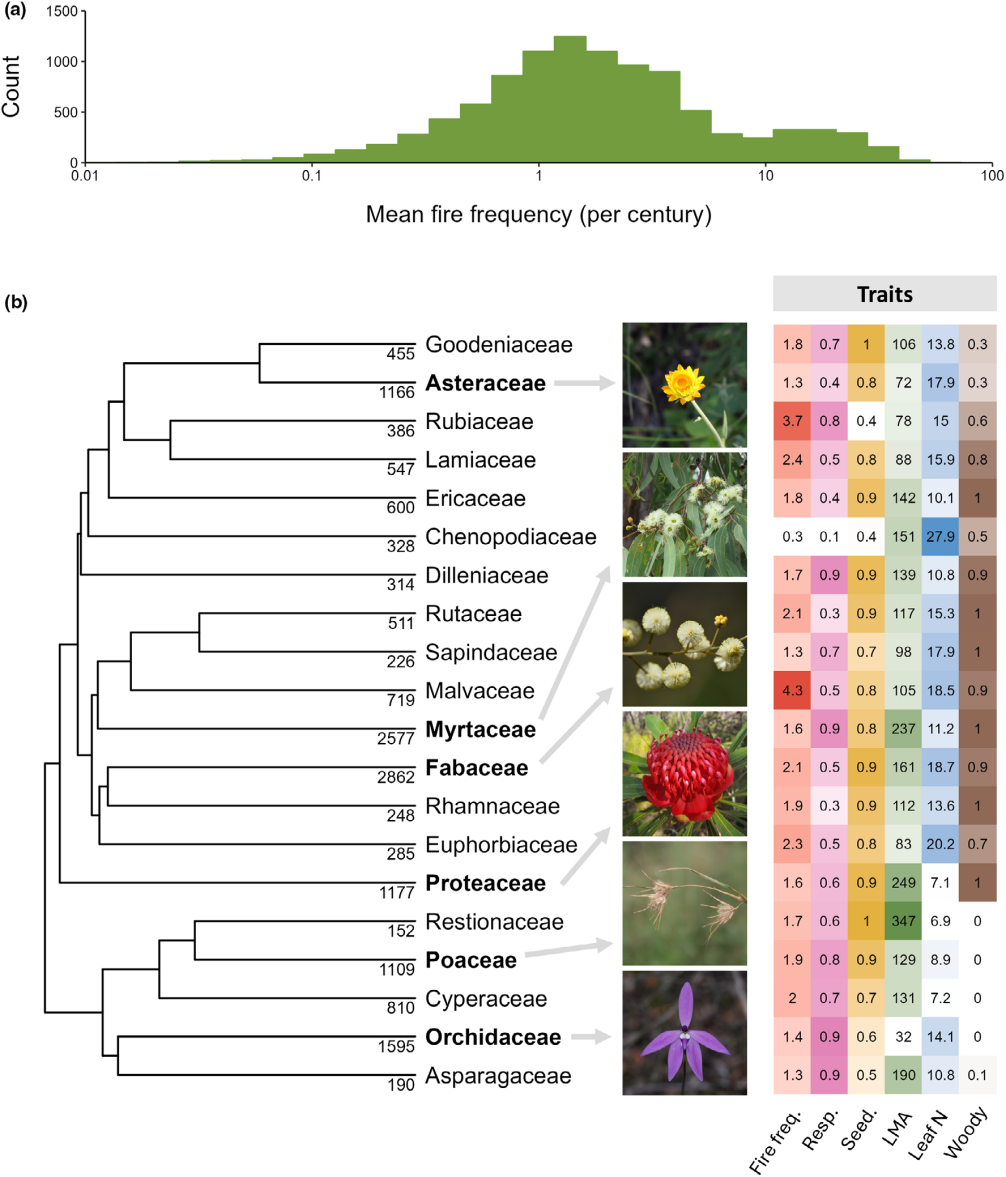
Fire frequencies experienced by plants across Australia, with (a) distribution of mean fire frequencies (per century) experienced by each species, and (b) Australian plant families with the highest coverage of resprouting data, showing fire response and leaf traits in relation to median fire frequency. Native species diversity of each family is indicated below each branch tip, with the top six most speciose families in bold. Example species are indicated by grey arrows: from top to bottom, they are: *Xerochrysum bracteatum* (@gregtasney (CC‐BY‐SA)), *Eucalyptus tetrodonta* (@nawocnai (CC‐BY‐NC)), *Acacia terminalis* (@tayloredtotayler (CC‐BY‐NC)), *Telopea speciosissima* (@vix_ (CC‐BY‐NC)), *Themeda triandra* (@arista_botha (CC‐BY‐NC‐SA)) and *Glossodia major* (@jimstone54 (CC‐BY‐NC)) (discoverable on iNaturalist). Traits are summarised and coloured for each family on the right (darker colours indicate higher values). Median fire frequency per century was calculated as the median of all species‐level mean fire frequencies in a given family. Mean fire frequencies for each species were calculated from the mean number of fires across a species' range from MODIS data (2000–2022). Fire response traits were calculated by finding the proportion of resprouters and postfire seeders, respectively, in each family. Median leaf mass per area (LMA; g m^−2^) and leaf nitrogen (N; mg g^−1^) were calculated as the mean LMA and leaf N per species and then finding the median of all species in the family. The tree was built with a dated phylogeny for seed plants by Smith & Brown (2018).

The average fire frequencies experienced by species in the dataset, across their known distribution, ranged from 0.01 fires per century to 53.3 fires per century, while the median fire frequency across all species was 1.6 fires per century (Fig. 2a). The median fire frequencies of the six most speciose Australian plant families (Fig. 2b, in bold) ranged from 2.1 fires per century for Fabaceae to 1.3 fires per century for Asteraceae. Out of the 20 Australian plant families with the highest coverage of resprouting data, median fire frequencies ranged from 0.3 (Chenopodiaceae) to 4.3 (Malvaceae) across families (Fig. 2b). These encapsulate the diversity of fire frequencies that exist across Australia.

### Fire response strategies, growth forms and life history

Out of 6387 woody species, 4022 were resprouters and 2254 were nonresprouters. 2092 woody species were documented as postfire seeders and 569 as nonseeders. 1672 species were capable of both resprouting and seeding, that is, facultative species. In 4634 total herbaceous species, 3006 were resprouters and 1599 were nonresprouters.

There were 683 annual herbaceous species and 3544 perennial herbaceous species. There were seven ‘woody’ species assigned as ‘annuals’, but these were species that could be classified as either woody or herbaceous. Within annual herbaceous species, 57 out of 680 with data (8.4%) were resprouters and 105 out of 111 with data (94.6%) were seeders. Within perennial herbaceous species, 2690 out of 3529 with data (76.2%) were resprouters and 530 out of 718 with data (73.8%) were seeders.

### Variation of fire response strategies and fire frequency in infraspecies

Where there were data on resprouting for both species and infraspecific levels, 12.8% of infraspecies were assigned a different value to their species counterparts. For postfire seeding, 18.3% of infraspecies were assigned differently. There was also variation in the fire frequencies experienced by infraspecies compared with species (Fig. S3).

### Relationships between fire response strategies and fire frequency

**Fig. 3:**
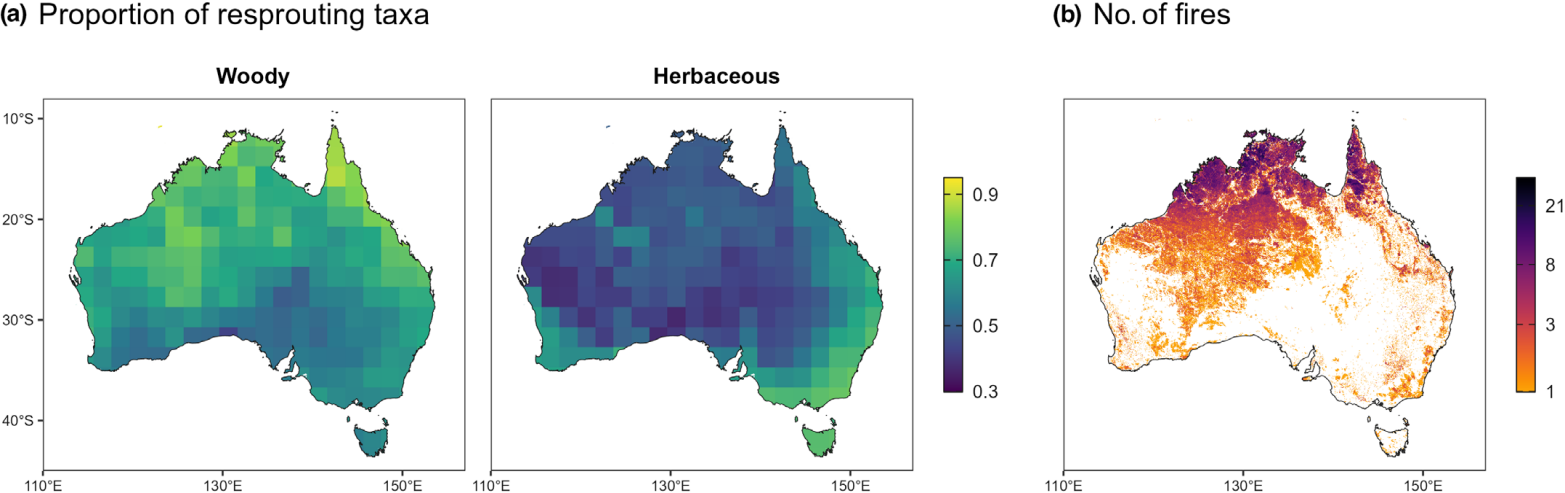
Mapping the distribution of fire response strategies at continental scale, with (a) proportions of resprouters in woody and herbaceous species across Australia and (b) the number of fires that occurred during the Moderate Resolution Imaging Spectroradiometer (MODIS) burnt area product (MCD64A1) dataset period (2000–2022; white denotes zero fires), at 500‐m resolution. Postfire seeding was excluded due to lack of data.

**Fig. 4:**
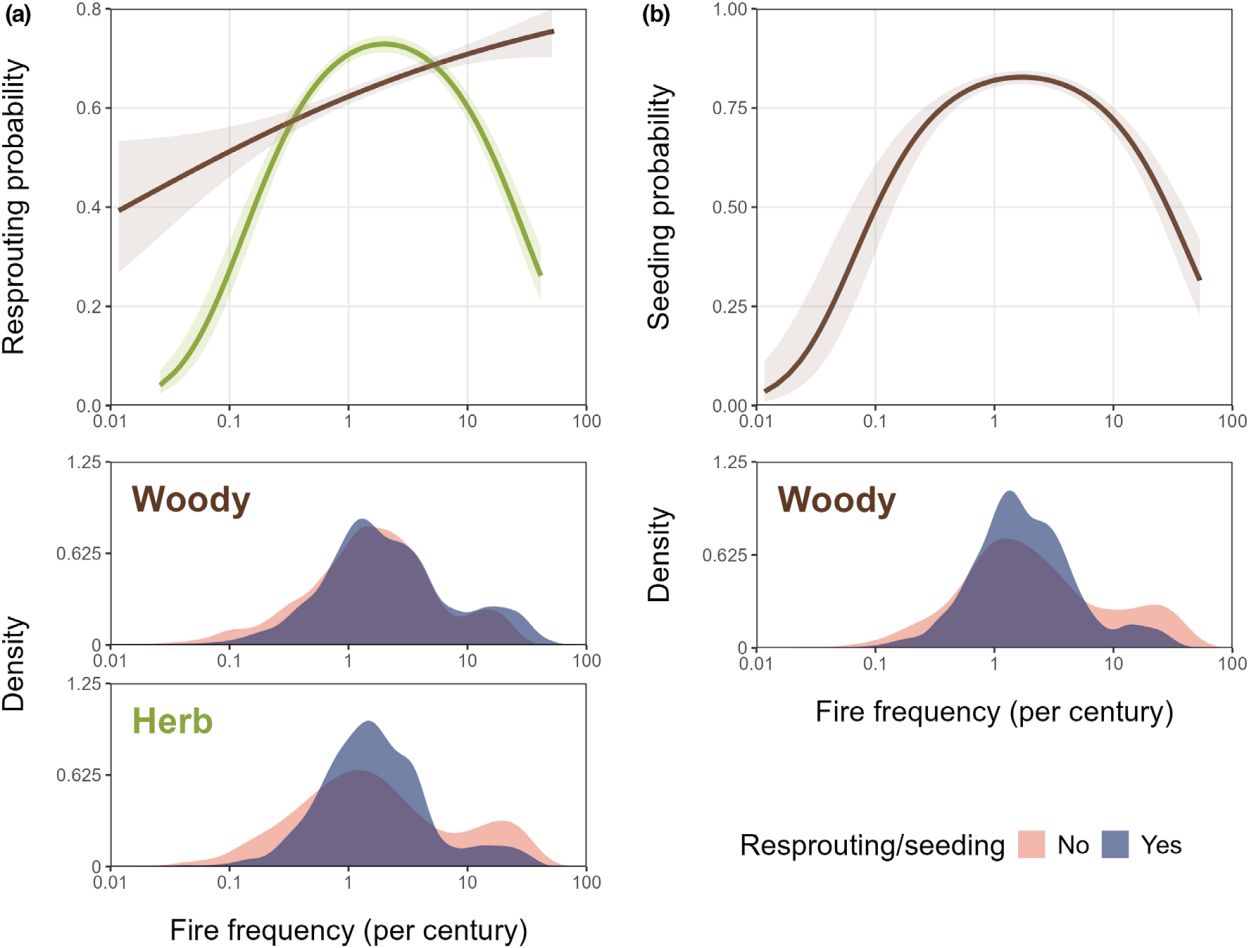
Observed changes in the frequency of two fire‐adapted traits with disturbance regime, as indicated by fire frequency. The lines in the upper panels show the modelled changes in the proportion of species with either (a) resprouting or (b) postfire seeding ability against fire frequency (per century), for woody (brown) and herbaceous (green) species, with 95% confidence intervals. Herbaceous species were omitted from (b) due to insufficient data. Statistics for these lines are in Tables 1 and 2; see text for number of observations in each group. Lower panels show the density functions for the number of woody and herbaceous species with and without these adaptations, along the gradient. Fire frequency is log‐scaled and represents the mean fire frequency across a species' range from 2000 to 2022 (see main text for details).

In woody species, the proportion of resprouters was highest in frequently burnt systems, particularly in northern Australia (Fig. 3). In herbaceous species, there were higher proportions of resprouters in south‐eastern and south‐western Australia, where fires are moderately frequent (Fig. 3). The probability of resprouting tends to increase with fire frequency for both woody and herbaceous species (Fig. 4a; Table 1). However, resprouting probability peaks at intermediate fire frequencies for herbaceous species, while resprouting probability continues to increase at high fire frequencies for woody species (Fig. 4a; Table 1). Nonetheless, resprouting is still a viable strategy for herbs at high fire frequencies, as > 20% of species still exhibit this strategy (Fig. 4a; Table 1). Resprouting is much less likely for herbs than for woody species at very low fire frequencies (Fig. 4a; Table 1). These relationships were also reproduced when phylogeny was taken into account, although with a slight decline in resprouting probability for woody plants at the highest fire frequencies (Fig. S4a; Table S2). There was no difference in outcomes when run with or without infraspecific taxa (Table S3), nor when run with or without annuals (Table S4).

**Table 1:** Association of (a) resprouting and (b) postfire seeding (both coded as TRUE or FALSE) with mean fire frequency (per century), and separate models run for each trait.

Predictors	Coefficient	CI	Odds ratios	CI	*P*
*(a) Resprouting*					
(Intercept)	0.88	0.81 to 0.96	2.42	2.24 to 2.62	**< 0.001**
Mean fires (log_10_)	0.70	0.55 to 0.86	2.01	1.73 to 2.36	**< 0.001**
(Mean fires (log_10_))^2^	−1.17	−1.33 to −1.01	0.31	0.26 to 0.36	**< 0.001**
Woody or herb (woody)	−0.38	−0.48 to −0.28	0.68	0.62 to 0.76	**< 0.001**
Mean fires (log_10_) × Woody or herb (woody)	−0.28	−0.47 to −0.09	0.76	0.62 to 0.92	**0.005**
(Mean fires (log_10_))^2^ × Woody or herb (woody)	1.13	0.94 to 1.33	3.11	2.56 to 3.79	**< 0.001**
Observations	10 012				
*R* ^2^ Tjur	0.031				
*(b) Postfire seeding*					
(Intercept)	1.55	1.42 to 1.68	4.54	4.05 to 5.11	**< 0.001**
Mean fires (log_10_)	0.66	0.37 to 0.95	1.62	1.26 to 2.07	**< 0.001**
Mean fires (log_10_)^2^	−1.22	−1.49 to −0.96	0.35	0.28 to 0.44	**< 0.001**
Observations	2576				
*R* ^2^ Tjur	0.039				

Values show fitted parameters and odds ratios of the response for a change in each predictor, with respective 95% confidence intervals (CI) in the following columns, from generalised linear models with a logit link and binomial response. *P*‐values < 0.05 are bolded. The odds ratio is the ratio of the probability of responding to the probability of not responding for a unit change in the predictor. Values < 1 indicate a lower likelihood of responding compared with not responding with an increase in the predictor. We also included growth form (woody or herbaceous) as a predictor for resprouting ability, but not for postfire seeding due to lack of data. Mean fire frequency was log‐transformed to reduce skewness. The number of observations and *R* ^2^ Tjur value are listed. The *R* ^2^ Tjur value, or the coefficient of discrimination, is the absolute value of the difference between the mean fitted probability for the TRUE response outcome and the mean fitted probability for the FALSE response outcome.

In woody species, the probability of postfire seeding is high at intermediate fire frequencies, decreasing dramatically with more frequent fires and very infrequent fires (Fig. 4b; Table 1). This unimodal relationship was also seen when accounting for phylogeny (Fig. S4b; Table S2) and when run with or without infraspecific‐level taxa (Table S3).

### Relationships between fire response strategies and leaf traits

**Fig. 5:**
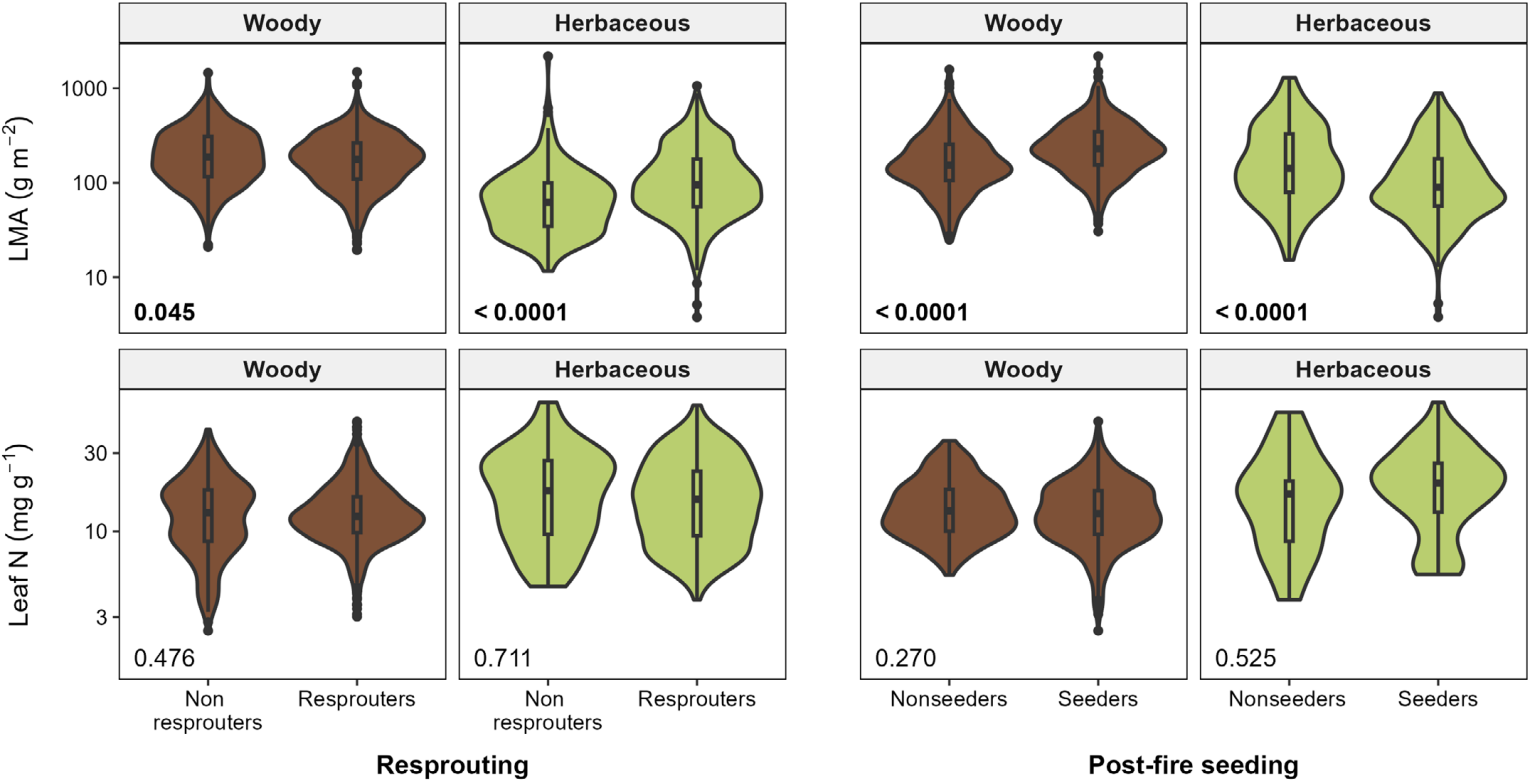
Comparing distributions of two leaf traits, leaf mass per area (LMA) and leaf nitrogen (N) content, among fire‐adapted and nonfire‐adapted species, within woody (brown) and herbaceous (green) species. Coloured areas show the density distribution of values across all species in each group, with boxplots showing the median, the first and third quartiles (hinges) and the largest/smallest value no further than 1.5 times the interquartile range (whiskers). Outliers (values outside whiskers) are plotted as individual points. *P*‐values indicate results of within group comparisons, adjusted with the Tukey method (see Table 2 for details).

In woody species, there was moderate evidence that LMA (log‐transformed) is slightly lower in resprouters than in nonresprouters (estimate = −0.041, df = 2836, *P* = 0.045; Fig. 5; Table 2). However, according to the phylogenetic regression, there was very strong evidence that the opposite is true; that is, woody resprouters have slightly higher LMA than nonresprouters (estimate = 0.031, df = 2370, *P* = <0.001; Table S5). There was very strong evidence that LMA is higher in seeders than in nonseeders (estimate = 0.147, df = 1494, *P* = <0.0001; Fig. 5; Table 2).

**Table 2:** Association of (a, b) mean leaf mass per area (LMA) (−g m^−2^) and (c, d) mean leaf nitrogen (N) content (mg g^−1^) with resprouting and postfire seeding, including woody or herbaceous growth form as an explanatory factor and any interactions.

	Estimates	CI	*P*
*(a) Mean LMA (log*_*10*_ *) vs resprouting*			
(Intercept)	1.79	1.75 to 1.84	**< 0.001**
Resprouting	0.20	0.15 to 0.25	**< 0.001**
Woody or herb (linear)	0.47	0.42 to 0.52	**< 0.001**
Resprouting × Woody or herb (linear)	−0.24	−0.30 to −0.18	**< 0.001**
Observations	2840		
*R* ^2^/*R* ^2^ adjusted	0.171/0.170		
*(b) Mean LMA (log*_*10*_ *) vs seeding*			
(Intercept)	2.08	2.01 to 2.14	**< 0.001**
Seeding	−0.18	−0.25 to −0.10	**< 0.001**
Woody or herb (linear)	0.02	−0.05 to 0.10	0.584
Seeding × Woody or herb (linear)	0.32	0.24 to 0.41	**< 0.001**
Observations	1498		
*R* ^2^/*R* ^2^ adjusted	0.162/0.161		
*(c) Mean leaf N content (log*_*10*_ *) vs resprouting*			
(Intercept)	1.21	1.16 to 1.26	**< 0.001**
Resprouting	−0.03	−0.09 to 0.03	0.287
Woody or herb (linear)	−0.13	−0.18 to −0.07	**< 0.001**
Resprouting × Woody or herb (linear)	0.05	−0.01 to 0.11	0.118
Observations	1636		
*R* ^2^/*R* ^2^ adjusted	0.025/0.023		
*(d) Mean Leaf N content (log*_*10*_ *) vs seeding*			
(Intercept)	1.17	1.09 to 1.27	**< 0.001**
Seeding	0.07	−0.03 to 0.18	0.174
Woody or herb (linear)	−0.04	−0.13 to 0.06	0.416
Seeding × Woody or herb (linear)	−0.10	−0.21 to 0.01	0.068
Observations	869		
*R* ^2^/*R* ^2^ adjusted	0.029/0.026		

Values are fitted parameters and 95% confidence intervals (CI) from linear models. *P*‐values < 0.05 are bolded. Number of observations and *R* ^2^ values are listed below each model.

In herbaceous species, there was very strong evidence that resprouters have higher LMA than nonresprouters (estimate = 0.201, df = 2836, *P* = <0.0001; Fig. 5; Table 2), including when annuals were removed (estimate = 0.117, df = 2746, *P* = 0.001; Fig. S5; Table S6). However, there was only little evidence of this from the phylogenetic regression (estimate = 0.102, df = 2370, *P* = 0.147; Table S5). There was very strong evidence that postfire seeders have lower LMA than nonseeders (estimate = −0.177, df = 1494, *P* = <0.0001; Fig. 5; Table 2), as well as when annuals were removed (estimate = −0.152, df = 1257, *P* = 0.004; Table S6). In both woody and herbaceous species, there was no evidence that leaf N content is different between resprouters and nonresprouters or between seeders and nonseeders (Fig. 5; Table 2).

## Discussion

Australia captures a broad range of vegetation types and fire frequencies, with species mean fire frequencies ranging from 0.01 to 53.3 fires per century. This range reflects those experienced globally. We found that resprouting probability was highest in areas with intermediate fire frequencies (1–5 fires per century; Murphy *et al*., 2013) for herbaceous plants, while resprouting probability continually increases with fire frequency for woody plants. Postfire seeding is most dominant at intermediate fire frequencies for woody plants. These findings provide the largest empirical support to date for long‐held concepts in the literature, that resprouting is inviable when disturbances are too frequent relative to productivity and that postfire seeding in woody plants is constrained by immaturity risk and senescence risk (Hilbert, 1987; Bellingham & Sparrow, 2000; Pausas, 2001; Bond & Midgley, 2003; Klimešová & Klimeš, 2003). Among woody species, we did not find support for the predicted decline in resprouting ability at the highest fire frequencies, but this is likely attributed to lower fire severity in these frequently burnt areas (Williams *et al*., 1998; Archibald *et al*., 2013; Murphy *et al*., 2013). Relationships were largely preserved when accounting for phylogenetic relationships, suggesting that patterns of fire response strategies and fire frequency are occurring across unrelated species.

The hypothesised hump‐shaped relationship between resprouting and fire frequency described by Bellingham & Sparrow (2000) for woody plants was found to also adequately describe the relationship for herbaceous plants (Fig. 4a). This was supported even when annual species were removed, suggesting that resprouting is less likely at high fire frequencies even among perennial species. Resprouting is hypothesised to be less energetically viable at high fire frequencies (Grady & Hoffmann, 2012; Fairman *et al*., 2019; Simpson *et al*., 2021). However, it is uncertain whether this explains the decline of resprouting in Australia's northern savannas. There, burning typically occurs during the dry season, when most plants will either die off or become dormant, irrespective of fire (Mott *et al*., 1985; Williams & Cook, 2001). Hence, perennial herbs that resprout from dormant basal buds are unlikely to lose additional aboveground biomass to fire. Potentially, if there is an increased probability of resprouting failure with the combined stressors of seasonal drought and frequent fire (Nolan *et al*., 2021), selection may act towards seeding. Correspondingly, Australia's northern savannas have a high richness of annual seeders (Andrew & Mott, 1983). However, resprouting in these areas is still relatively important, and herbs that do resprout, do so from underground organs, or basal meristems in the case of grasses (Mott *et al*., 1985; Simpson *et al*., 2021). In intermediate fire frequencies, herbaceous resprouters outcompete seeders as they can quickly re‐establish postfire using energy reserves (Zimmermann *et al*., 2008; Simpson *et al*., 2021). This is seen in the high proportions of herbaceous resprouters in south‐eastern Australia where fires are moderately frequent (Pausas & Bradstock, 2007; Hammill *et al*., 2016; Fig. 3). At low fire frequencies, resprouting probability decreases, likely dueF to the high costs of maintaining buds when fires are rare (Simpson *et al*., 2021). For example, Asteraceae, a primarily herbaceous family, occurs predominantly in seldom burnt environments (median fire frequency per century = 1.3) and has a relatively low proportion of resprouters (44%; Fig. 2b). Our results examine a much wider range of fire frequencies compared with Simpson *et al*. (2021), but in the areas of overlap, our results for all herbaceous species are consistent with those they found for Poaceae.

Woody species exhibit a similar relationship to herbaceous species, where resprouting probability increases with fire frequency (Fig. 4a); however, unlike herbaceous species, resprouting probability does not decline at very high fire frequencies, or declines only slightly according to the phylogenetic analysis (Fig. S4a). Resprouting probability is highest for woody species in frequently burnt systems, evidenced in the high proportion of woody resprouters across northern Australia, compared with herbaceous resprouters (Fig. 3a). This may seem inconsistent with the decline in resprouting at high disturbance frequencies predicted by Bellingham & Sparrow (2000); however, their model assumed high disturbance severity. Places with frequent fire are fundamentally constrained to moderate maximum fire intensities (Archibald *et al*., 2013), and the proportion of species that resprout is typically higher as disturbance severity decreases (Bellingham & Sparrow, 2000; Vesk & Westoby, 2004). Fire regimes in the savannas of northern Australia are characterised by frequent, low‐ to moderately high‐intensity surface fires (Williams *et al*., 1998; Archibald *et al*., 2013; Murphy *et al*., 2013), which woody species typically survive with a combination of thick bark and resprouting mechanisms (Barlow *et al*., 2003; Lawes *et al*., 2011a,b). The majority of trees in these savannas resprout epicormically (Clarke *et al*., 2015), unless top‐killed (more common in juvenile trees), to which basal resprouting is the dominant response (Werner & Franklin, 2010; Lawes *et al*., 2011a). This could explain the continual increase in resprouting probability even at the highest fire frequencies. At low fire frequencies, found in both the arid and wet extremes of Australia, resprouting is less likely and may have evolved in response to disturbances other than fire (Pausas & Keeley, 2014b; Pausas *et al*., 2016). Resprouting in these mesic systems is probably enabled by higher productivity, compared with their arid counterparts (Bellingham & Sparrow, 2000), as seen in slightly higher resprouting proportions in temperate Australia compared with the largely unburnt arid interior (Fig. 3).

Postfire seeding displayed the characteristic hump‐shaped relationship with fire frequency for woody species (Fig. 4b). This affirms that many woody seeders are constrained by ‘immaturity risk’, where seeders are unable to reach reproductive maturity if fire frequency is too high (Keeley *et al*., 1999), and ‘senescence risk’, where seeders are unable to produce a new cohort of seedlings when FRIs are longer than the longevity of the seed bank (Keeley, 1986). As young plants are more vulnerable to fire than adults (the majority of aboveground biomass being exposed), the persistence of woody seeders is dependent on sufficient fire‐free periods during which plants can reach reproductive maturity from seed, grow to heights that escape the flame zone (Lawes *et al*., 2011a; Bond *et al*., 2012), or develop thicker bark and/or resprouting ability while still a seedling or juvenile, as is the case for facultative resprouters in Australia's northern savannas (Lawes *et al*., 2011a). While woody seeders are restricted by ‘immaturity risk’ in environments that burn frequently, herbaceous seeders are generally able to reach reproductive maturity much more rapidly and thus some can persist even in FRIs as short as 1 yr (e.g. grass annual seeders; Mott *et al*., 1985; Werner & Franklin, 2010; Simpson *et al*., 2021). Interestingly, there are some woody seeding species that evolve to dramatically reduce their time to maturity and may eventually be considered herbaceous, such as *Androcalva rosea*, which can reproduce and senesce in < 16 months, despite its sub‐shrub growth form (Bell & Copeland, 2004). However, immaturity risk is a species‐specific concept, where shortening FRIs are likely detrimental to a species irrespective of whether it is adapted to high, moderate or low fire frequencies. In general, woody obligate seeders have longer maturity ages than herbaceous seeders (Smith & Donoghue, 2008), explaining the decline of woody seeders at high fire frequencies. Although herbaceous seeders are less constrained by high fire frequencies, they are also expected to decline at lower fire frequencies due to high competition and senescence risk (Zimmermann *et al*., 2008; Pausas & Keeley, 2014b; Simpson *et al*., 2021), but this remains untested.

In order to avoid immaturity risk, seeders were hypothesised to adopt a fast‐growing strategy along the leaf economics spectrum, expressed by a lower investment in each leaf (lower LMA), high nutrient contents (higher leaf N) and high photosynthetic rates (Wright *et al*., 2004). This was supported by our results for herbaceous species, including strong evidence that herbaceous resprouters have higher LMA than herbaceous nonresprouters, and herbaceous seeders have lower LMA than herbaceous nonseeders (Fig. 5). This was also the case among just perennials (Fig. S5; Table S6). Herbaceous species seem adequately described by the hypothesis that these two fire response strategies have contrasting life histories: one that avoids immaturity risk through a faster growth strategy and another that is slower growing and prioritises resource retention in between fires (Pausas *et al*., 2004; Pausas & Keeley, 2014a).

In woody species, there was little evidence that seeders adopt a faster‐growing leaf economics strategy than resprouters. Instead, we found very strong evidence that LMA is actually higher in seeders than nonseeders, suggesting that at least for some woody seeders, a resource‐retaining strategy (higher LMA, lower leaf nutrients) between fires may be more important than fast growth to reduce immaturity risk. This is especially important where drought is recurrent, a common event across large areas of the Australian continent (Jiang *et al*., 2017) and likely experienced by woody seeders that dominate at intermediate fire frequencies (Fig. 4b). Many obligate seeders in Mediterranean climates have been found to be drought tolerant, associated with functional traits including longer leaf lifespan, higher LMA and lower leaf turgor loss point (Ackerly, 2004; Paula & Pausas, 2006; Saura‐Mas & Lloret, 2007). This resource‐conserving strategy is exhibited in the woody family Proteaceae, which has higher average LMA and lower leaf N than most other Australian plant families (Fig. 2b; Cornwell *et al*., 2014). Additionally, leaf functional traits may vary throughout an individual's lifespan (Henn & Damschen, 2021; Westoby *et al*., 2022). A seeder may prioritise growth at early life stages to establish quickly postfire, but later shift to a more resource‐conservative strategy (Mason *et al*., 2013; Funk *et al*., 2021). Slow‐growing woody seeders may be particularly vulnerable to immaturity risk under increasing fire frequencies (Bradstock & Kenny, 2003; Kraaij *et al*., 2013).

This analysis was at the species and infraspecific level, but in response to shifting fire regimes, there may also be changes within species or within infraspecies to fire adaptation. Even in our dataset, we found differences in trait values, as well as the mean fire frequencies experienced, between infraspecies and their species counterparts. Resprouting and seeding are not mutually exclusive fire response strategies; they are coexisting approaches, which have shifting proportions along different gradients of fire regime characteristics. The viability of both strategies across much of the fire frequency gradient also explains the high proportion of facultative species (species able to both resprout and seed) in Australia overall (1672 species or 15% of the dataset was facultative), and populations of these species may be under shifting evolutionary pressures throughout their ranges.

Fire frequencies have never been static through geological time, and humans have changed fire regimes through increased ignitions, and more recently, fire suppression and climate change (Krawchuk *et al*., 2009; Pausas & Keeley, 2009; Andela *et al*., 2017; Balch *et al*., 2017). Moreover, European practices in Australia with regard to fire are fundamentally different from those of the Traditional Owners (Perry *et al*., 2018). We acknowledge that a key limitation to using data from the MODIS satellite is its short snapshot of 22 yr. While fire frequencies in that 22‐yr period may not reflect preceding periods, the MODIS data have been used (with even shorter periods) to detect large‐scale differences in fire regimes (Bond & Midgley, 2012). The 22‐yr period is likely sufficient to distinguish very fire‐prone environments from infrequently burnt environments, and for a successful ranking of mean fire frequencies across species. Because mean fire frequency was calculated across each species' range, we expect that a species with more unburnt points than another will effectively rank the species as more fire‐sensitive than species with less unburnt points. Additionally, species occurring in mostly or all unburnt cells, often also occurring in few cells overall, were excluded (157 species). Because of the ubiquity of fire across the Australian continent, only 1.1% of species in our dataset occurred in entirely unburnt cells, after filtering species occurring in < 10 pixels. The combination of modern fire history and paleofire reconstruction data where available could further test the relationships found in this study (Marlon, 2020).

Large‐scale relationships of fire response strategies and fire regime characteristics are common ecological hypotheses (Bellingham & Sparrow, 2000; Bond & Midgley, 2003; Pausas & Keeley, 2014a) for which sufficient data to be empirically tested have become available only recently. Our findings are one of the first to provide a broad overview of how fire response strategies sort along a fire frequency gradient, using large‐scale, real‐world data. Our work is timely, as fire regimes are currently being altered due to climate change and other human‐influenced drivers (Hardesty *et al*., 2005; Pausas & Keeley, 2014b; Andela *et al*., 2017; Canadell *et al*., 2021), potentially leading to shifts in the distribution of each fire response strategy and the modification, expansion and contraction of various vegetation communities (Nolan *et al*., 2021). Slow‐growing obligate seeders are particularly vulnerable to increasing fire frequencies when paired with warmer and drier conditions, which limit plant growth and hence diminish the fire interval window required for their persistence (‘interval squeeze’; Enright *et al*., 2015). Predicting vegetation responses to shifting fire regimes needs to be underpinned by both clear theory and robust empirical analyses; our results add continental‐scale empirical data to this effort.

## Competing interests

None declared.

## Author contributions

SY, WC, MKJO and DSF designed the study. SY extracted and analysed the data. SY, WKC, MKJO and DSF interpreted the data. SY wrote the manuscript with help from all the authors.

## Disclaimer

The New Phytologist Foundation remains neutral with regard to jurisdictional claims in maps and in any institutional affiliations.

## Supporting information


**Fig. S1** Comparison of two methods for estimating average fire frequency for an example species.
**Fig. S2** Comparison of average fire frequencies estimated using mean event rate and survival analysis methods.
**Fig. S3** Differences in mean fire frequency (per century) experienced by infraspecies vs their species counterparts.
**Fig. S4** Relationships between resprouting and seeding vs fire frequency per century, as predicted by phylogenetic logistic regression.
**Fig. S5** Comparison of leaf mass per area among resprouting and nonresprouting herbaceous species, grouped by annual/perennial life history.
**Table S1** Woodiness trait values from AusTraits divided into woody, herbaceous, semi‐woody and ambiguous categories.
**Table S2** Association of resprouting and seeding with fire frequency and woody or herbaceous growth form, as fit by phylogenetic logistic regression.
**Table S3** Association of resprouting and seeding with fire frequency and woody or herbaceous growth form, for species‐level taxa only.
**Table S4** Association of resprouting with fire frequency and woody or herbaceous growth form, with annuals excluded.
**Table S5** Association of leaf mass per area and leaf nitrogen content with resprouting and postfire seeding, fit by phylogenetic linear regression.
**Table S6** Association of leaf mass per area and leaf nitrogen content with resprouting and postfire seeding, with annuals excluded.
Please note: Wiley is not responsible for the content or functionality of any Supporting Information supplied by the authors. Any queries (other than missing material) should be directed to the *New Phytologist* Central Office.

## Data Availability

Data and R code are available on Zenodo: doi: 10.5281/zenodo.14751969.
