# Supplementary material for: Continental‐scale empirical evidence for relationships between fire response strategies and fire frequency
Source: New Phytol. 2025 Feb 11;246(2):528–42. doi: 10.1111/nph.20464 (PMC11923400; doi:10.1111/nph.20464)
Supplement: Supplementary file 1 — Fig. S1 Comparison of two methods for estimating average fire frequency for an example species. Fig. S2 Comparison of average fire frequencies estimated using mean event rate and survival analysis methods. Fig. S3 Differences in mean fire frequency (per century) experienced by infraspecies vs their species counterparts. Fig. S4 Relationships between resprouting and seeding vs fire frequency per century, as predicted by phylogenetic logistic regression. Fig. S5 Comparison of leaf mass per area among resprouting and nonresprouting herbaceous species, grouped by annual/perennial life history. Table S1 Woodiness trait values from AusTraits divided into woody, herbaceous, semi‐woody and ambiguous categories. Table S2 Association of resprouting and seeding with fire frequency and woody or herbaceous growth form, as fit by phylogenetic logistic regression. Table S3 Association of resprouting and seeding with fire frequency and woody or herbaceous growth form, for species‐level taxa only. Table S4 Association of resprouting with fire frequency and woody or herbaceous growth form, with annuals excluded. Table S5 Association of leaf mass per area and leaf nitrogen content with resprouting and postfire seeding, fit by phylogenetic linear regression. Table S6 Association of leaf mass per area and leaf nitrogen content with resprouting and postfire seeding, with annuals excluded. Please note: Wiley is not responsible for the content or functionality of any Supporting Information supplied by the authors. Any queries (other than missing material) should be directed to the New Phytologist Central Office. [file NPH-246-528-s001.pdf]

New Phytologist

***New Phytologist* Supporting Information**

Article title: Continental-scale empirical evidence for relationships between fire response strategies and fire frequency

Authors: Sophie Yang, Mark K. J. Ooi, Daniel S. Falster, William K. Cornwell

Article acceptance date: 23 January 2025

The following Supporting Information is available for this article:

Fig. S1 Comparison of two methods for estimating average fire frequency for an example species.

Fig. S2 Comparison of average fire frequencies estimated using mean event rate and survival analysis methods.

Fig. S3 Differences in mean fire frequency (per century) experienced by infraspecies versus their species counterparts.

Fig. S4 Relationships between resprouting and seeding versus fire frequency per century, as predicted by phylogenetic logistic regression.

Fig. S5 Comparison of leaf mass per area among resprouting and non-resprouting herbaceous species, grouped by annual/perennial life history.

Table S1 Woodiness trait values from AusTraits divided into woody, herbaceous, semi-woody and ambiguous categories.

Table S2 Association of resprouting and seeding with fire frequency and woody or herbaceous growth form, as fit by phylogenetic logistic regression.

Table S3 Association of resprouting and seeding with fire frequency and woody or herbaceous growth form, for species-level taxa only.

Table S4 Association of resprouting with fire frequency and woody or herbaceous growth form, with annuals excluded.

Table S5 Association of leaf mass per area and leaf nitrogen content with resprouting and post-fire seeding, fit by phylogenetic linear regression.

Table S6 Association of leaf mass per area and leaf nitrogen content with resprouting and post-fire seeding, with annuals excluded.

Fig. S1 Comparison of two methods for estimating average fire frequency, for one example species (*Acacia longifolia*): (1) mean event rate, and (2) survival analysis following methods by Simpson *et al.* (2021). Method 1 uses each occurrence point as a replicate while Method 2 uses multiple inter-fire intervals within an occurrence point as replicates. The histogram of number of fires in Method 1 represents actual raw data from MODIS while the histogram of inter-fire intervals (Method 2) represents randomly simulated data from a Weibull distribution of shape and scale derived from fitting the model. Data were simulated because actual data often contained censored time intervals due to the short length of the MODIS period. The sampling period of MODIS was 22.17 years. Species occurrences were extracted from the Global Biodiversity Information Facility (GBIF). Picture of *Acacia longifolia* is credited to Jonathan M (CC BY-NC) on iNaturalist.

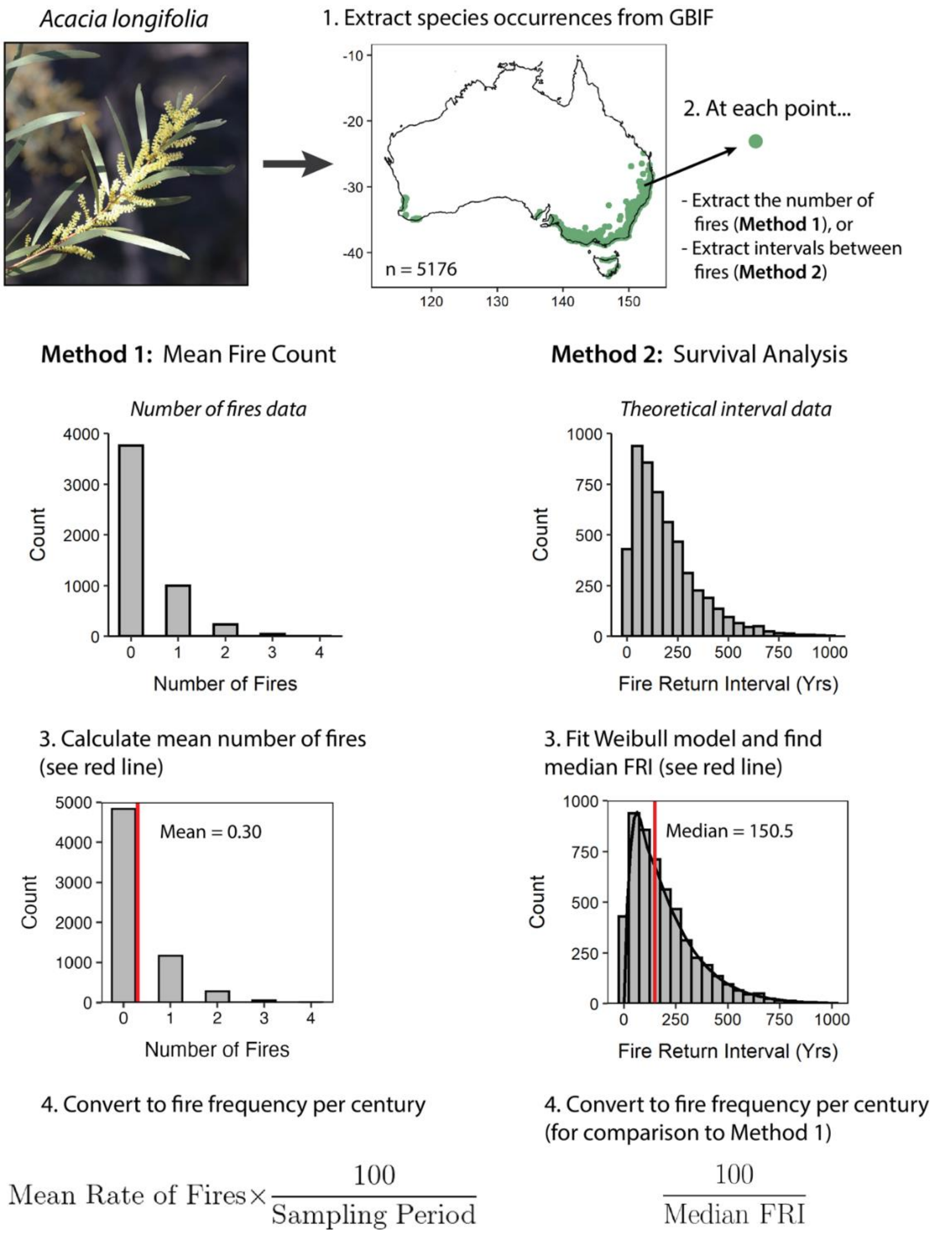

Fig. S2 Comparison of average fire frequencies (per century) of each species using mean event rate and survival analysis (Simpson *et al.*, 2021) methods (see main text for details), for woody and herbaceous plants.

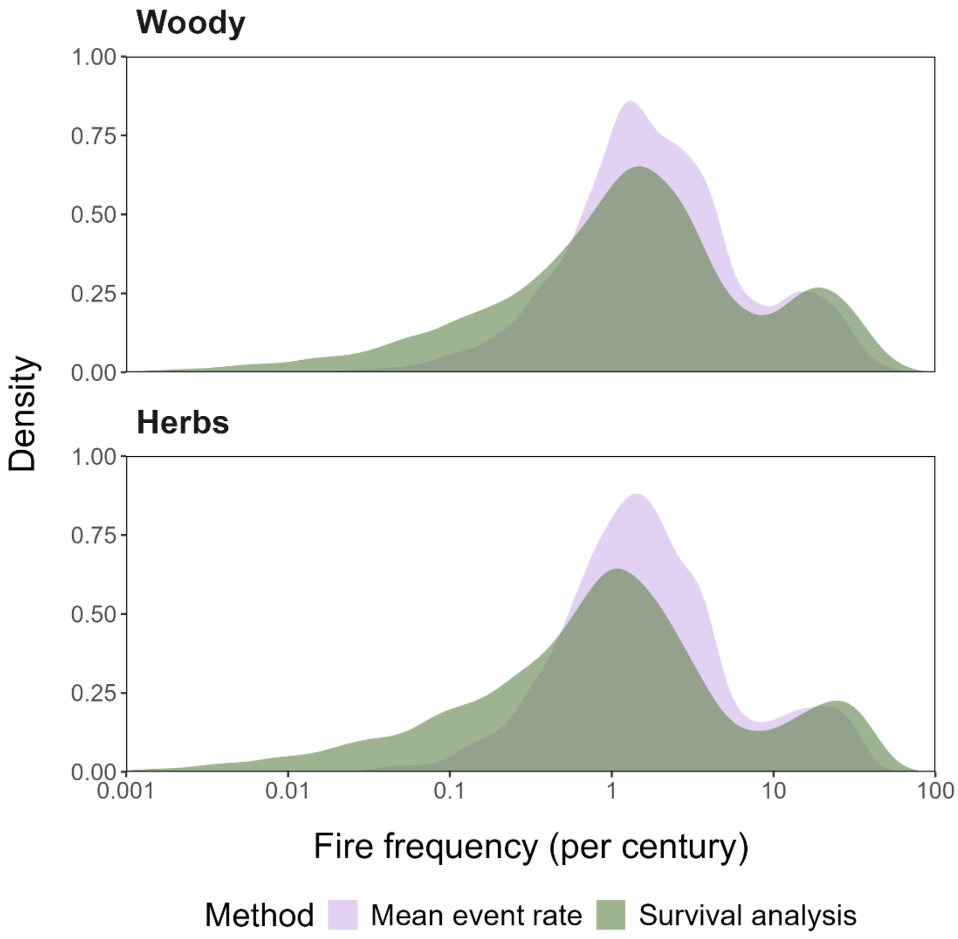

Fig. S3 Differences in mean fire frequency (per century) experienced by infraspecies versus their species counterparts. Mean fire frequencies for each taxon were the mean number of fires across a taxon's range from MODIS data (2000 to 2022).

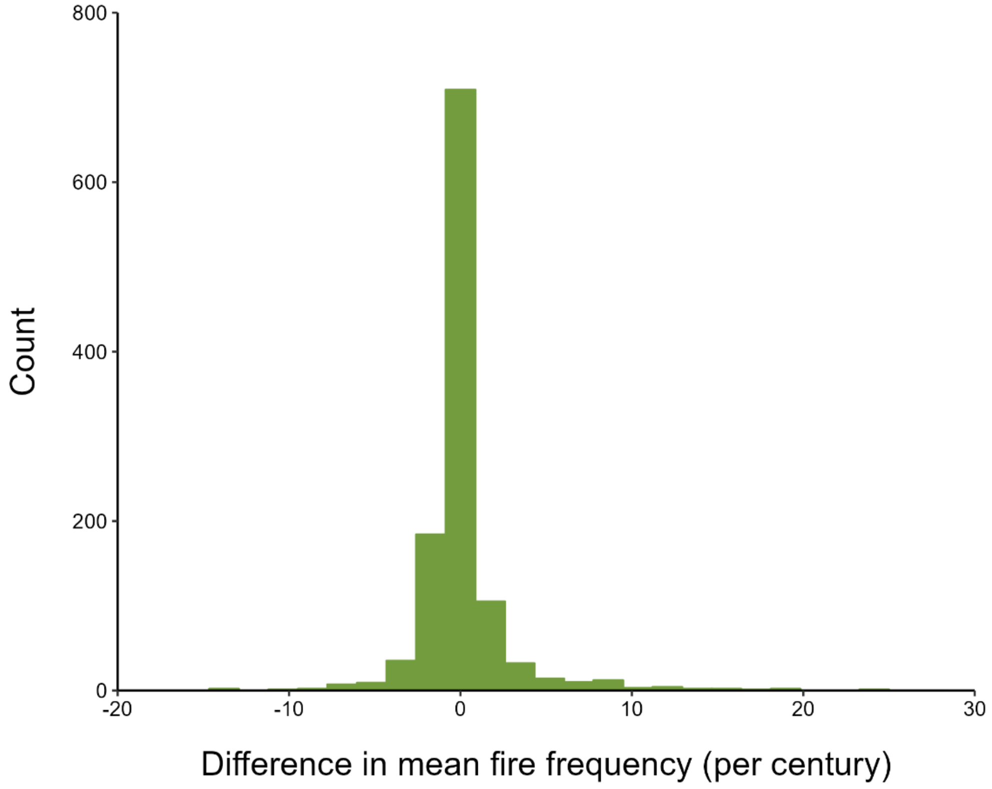

New Phytologist

Fig. S4 Observed changes in the frequency of two fire-adapted traits with disturbance regime, as indicated by fire frequency. Lines show changes in the proportion of species with either (a) resprouting or (b) post-fire seeding ability against fire frequency (per century), as modelled by phylogenetic logistic regression, for woody (brown) and herbaceous (green) species, with 95% confidence intervals. Herbaceous species were omitted from (b) due to insufficient data. Fire frequency is log-transformed and represents the mean fire frequencies across a species' range from 2000 to 2022 (see main text for details).

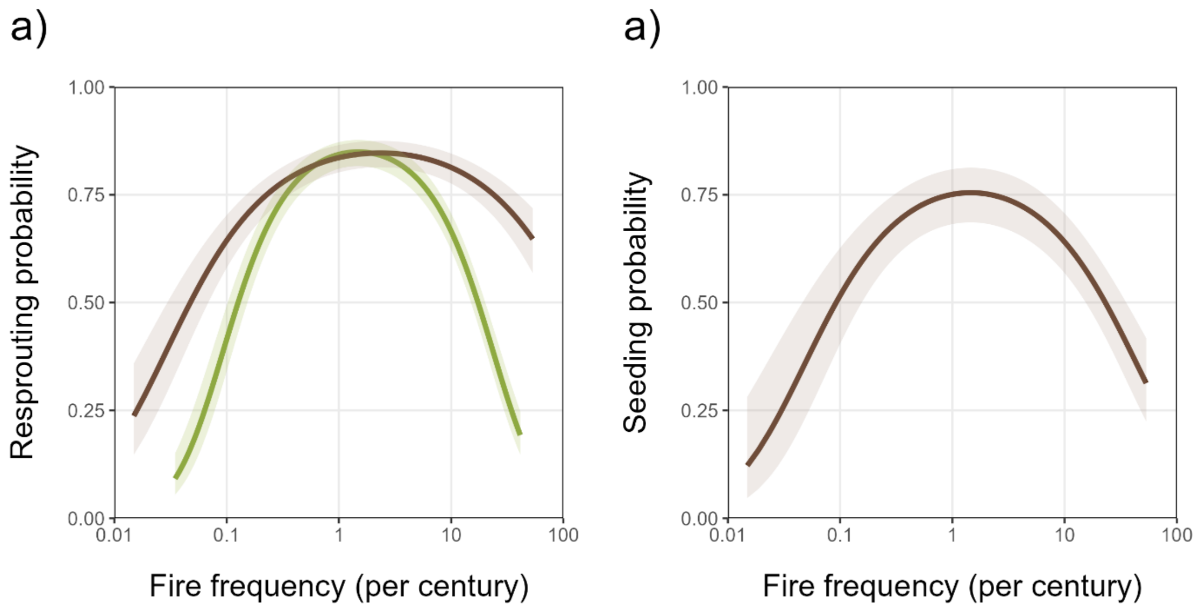

Fig. S5 Comparing distributions of leaf mass per area (LMA) among resprouting and non-resprouting herbaceous species, grouped by annuals and perennials. There were insufficient data to compare among seeders and non-seeders, or to compare leaf N. Coloured areas show the density distribution of values across all species in each group, with boxplots showing the median, the first and third quartiles (hinges) and the largest/smallest value no further than 1.5 times the interquartile range (whiskers). Outliers (values outside whiskers) are plotted as individual points.

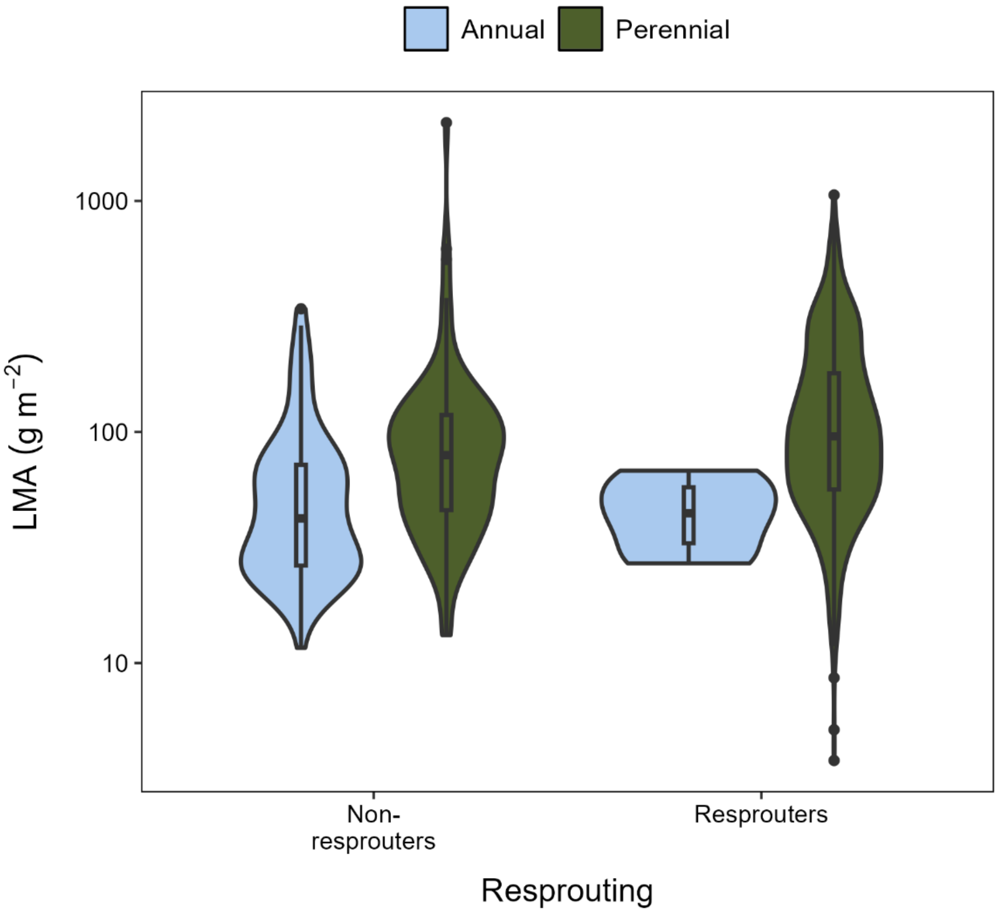

New Phytologist

Table S1 Woodiness (`woodiness_detailed`) values from the `Wenk_2022` dataset in AusTraits, simplified into woody, herbaceous, semi-woody and ambiguous categories.

Woody	Herbaceous	Semi-woody	Ambiguous
woody	herbaceous	semi_woody	woody_like_stem
semi_woody woody	herbaceous woody	semi_woody woody_base	woody_base
woody woody_base	herbaceous woody_base		
	herbaceous semi_woody		
	herbaceous semi_woody woody		

AusTraits records are sometimes combinations of allowed categorical values, e.g., species that exhibit both growth forms.

Table S2 Association of (a) resprouting and (b) post-fire seeding (both coded as TRUE or FALSE) with mean fire frequency (per century), as **fit by phylogenetic logistic regression** and with separate models run for each trait.

Predictors	*Coefficient*	*CI*	*Odds Ratios*	*CI*	*P*
**(a) Resprouting**					
(Intercept)	1.69	1.45 – 1.92	5.39	4.28 – 6.81	**<0.001**
Mean fires [log10]	0.52	0.34 – 0.69	1.68	1.41 – 2.00	**<0.001**
(Mean fires [log10])^2	-1.51	-1.72 – -1.30	0.22	0.18 – 0.27	**<0.001**
Woody or herb [woody]	-0.06	-0.30 – 0.19	0.95	0.74 – 1.21	0.658
Mean fires [log10] * Woody or herb [woody]	-0.08	-0.29 – 0.14	0.93	0.75 – 1.15	0.486
(Mean fires [log10])^2 * Woody or herb [woody]	0.92	0.67 – 1.16	2.50	1.96 – 3.19	**<0.001**
Observations	7465				
R^2 Tjur	0.031				
**(b) Post-fire seeding**					
(Intercept)	1.10	0.76 – 1.44	3.01	2.14 – 4.23	**<0.001**
Mean fires [log10]	0.26	0.04 – 0.47	1.29	1.04 – 1.61	**0.022**
(Mean fires [log10])^2	-0.78	-1.02 – -0.54	0.46	0.36 – 0.58	**<0.001**
Observations	2100				
R^2 Tjur	0.039				

Values show the fitted parameters and odds ratio of the response for a change in each predictor, with respective 95% confidence intervals (CI) in the following columns. P-values < 0.05 are bolded. The odds ratio is the ratio of the probability of responding to the probability of not responding for a unit change in the predictor. Values <1 indicate a lower likelihood of responding compared to not responding with an increase in the predictor. We also included growth form (woody or herbaceous) as a predictor for resprouting ability, but not for post-fire seeding due to lack of data. Mean fire frequency was log-transformed to reduce skewness. Number of observations and R^2 Tjur value are listed. The R^2 Tjur value, or the coefficient of discrimination, is the absolute value of the difference between the mean fitted probability for the TRUE response outcome and the mean fitted probability for the FALSE response outcome.

Table S3 Association of (a) resprouting and (b) post-fire seeding (both coded as TRUE or FALSE) with mean fire frequency (per century), and separate models run for each trait, for **species-level taxa only** (excluding below-species level taxa).

Predictors	*Coefficient*	*CI*	*Odds Ratios*	*CI*	*P*
**(a) Resprouting**					
(Intercept)	0.92	0.83 – 1.00	2.50	2.30 – 2.72	**<0.001**
Mean fires [log10]	0.68	0.51 – 0.84	1.97	1.67 – 2.32	**<0.001**
(Mean fires [log10])^2	-1.17	-1.34 – -1.01	0.31	0.26 – 0.37	**<0.001**
Woody or herb [woody]	-0.39	-0.50 – -0.28	0.68	0.61 – 0.76	**<0.001**
Mean fires [log10] * Woody or herb [woody]	-0.26	-0.47 – -0.05	0.77	0.63 – 0.95	**0.015**
(Mean fires [log10])^2 * Woody or herb [woody]	1.15	0.94 – 1.36	3.16	2.57 – 3.90	**<0.001**
Observations	8623				
R^2 Tjur	0.033				
**(b) Post-fire seeding**					
(Intercept)	1.54	1.42 – 1.67	4.67	4.13 – 5.29	**<0.001**
Mean fires [log10]	0.55	0.29 – 0.82	1.74	1.33 – 2.28	**<0.001**
(Mean fires [log10])^2	-1.15	-1.40 – -0.90	0.32	0.25 – 0.41	**<0.001**
Observations	2266				
R^2 Tjur	0.048				

Values show the fitted parameters and odds ratio of the response for a change in each predictor, with respective 95% confidence intervals (CI) in the following columns, from generalised linear models with a logit link and binomial response. P-values < 0.05 are bolded. The odds ratio is the ratio of the probability of responding to the probability of not responding for a unit change in the predictor. Values <1 indicate a lower likelihood of responding compared to not responding with an increase in the predictor. We also included growth form (woody or herbaceous) as a predictor for resprouting ability, but not for post-fire seeding due to lack of data. Mean fire frequency was log-transformed to reduce skewness. Number of observations and R^2 Tjur value are listed. The R^2 Tjur value, or the coefficient of discrimination, is the absolute value of the difference between the mean fitted probability for the TRUE response outcome and the mean fitted probability for the FALSE response outcome.

Table S4 Association of resprouting with mean fire frequency (per century), for **perennial species only**.

Predictors	*Coefficient*	*CI*	*Odds Ratios*	*CI*	*P*
(Intercept)	1.33	1.24 – 1.43	3.79	3.46 – 4.17	**<0.001**
Mean fires [log10]	0.45	0.27 – 0.62	1.56	1.31 – 1.86	**<0.001**
(Mean fires [log10])^2	-0.81	-0.99 – -0.63	0.44	0.37 – 0.53	**<0.001**
Woody or herb [woody]	-0.83	-0.94 – -0.71	0.44	0.39 – 0.49	**<0.001**
Mean fires [log10] * Woody or herb [woody]	-0.02	-0.23 – 0.19	0.98	0.79 – 1.21	0.842
(Mean fires [log10])^2 * Woody or herb [woody]	0.78	0.57 – 1.00	2.19	1.76 – 2.71	**<0.001**
Observations	9293				
R^2 Tjur	0.030				

Values show the fitted parameters and odds ratio of the response for a change in each predictor, with respective 95% confidence intervals (CI) in the following columns, from a generalised linear model with a logit link and binomial response. P-values < 0.05 are bolded. The odds ratio is the ratio of the probability of responding to the probability of not responding for a unit change in the predictor. Values <1 indicate a lower likelihood of responding compared to not responding with an increase in the predictor. We also included growth form (woody or herbaceous) as a predictor for resprouting ability. Mean fire frequency was log-transformed to reduce skewness. Number of observations and R^2 Tjur value are listed. The R^2 Tjur value, or the coefficient of discrimination, is the absolute value of the difference between the mean fitted probability for the TRUE response outcome and the mean fitted probability for the FALSE response outcome.

New Phytologist

Table S5 Association of (a,b) mean leaf mass per area (LMA) (g m^{-2}) and (c,d) mean leaf nitrogen (N) content (mg g^{-1}) with resprouting and post-fire seeding (both coded as TRUE or FALSE), including woody or herbaceous growth form as an explanatory factor and any interactions, **as fit from a phylogenetic linear model**.

	Estimates	*CI*	*P*
**(a) Mean LMA [log10] versus resprouting**			
(Intercept)	2.03	-1.64 – 5.69	0.278
Resprouting	0.10	0.01 – 0.19	**0.025**
Woody or herb [linear]	0.09	-0.05 – 0.22	0.200
Resprouting * Woody or herb [linear]	-0.06	-0.15 – 0.04	0.229
Observations	2374		
R^2 / R^2 adjusted	0.009/0.008		
**(b) Mean LMA [log10] versus seeding**			
(Intercept)	2.25	-0.16 – 4.66	0.067
Seeding	-0.19	-0.26 – -0.11	**<0.001**
Woody or herb [linear]	-0.13	-0.27 – 0.01	0.059
Seeding * Woody or herb [linear]	0.31	0.22 – 0.40	**<0.001**
Observations	1291		
R^2 / R^2 adjusted	0.033/0.031		
**(c) Mean leaf N content [log10] versus resprouting**			
(Intercept)	1.24	0.13 – 2.35	**0.028**
Resprouting	-0.02	-0.07 – 0.04	0.615
Woody or herb [linear]	-0.14	-0.21 – -0.07	**<0.001**
Resprouting * Woody or herb [linear]	0.01	-0.05 – 0.07	0.631
Observations	1373		
R^2 / R^2 adjusted	0.015/0.013		
**(d) Mean Leaf N content [log10] versus seeding**			
(Intercept)	1.13	0.02 – 2.25	**0.047**
Seeding	-0.02	-0.17 – 0.12	0.760
Woody or herb [linear]	-0.01	-0.17 – 0.14	0.865
Seeding * Woody or herb [linear]	-0.01	-0.16 – 0.14	0.901
Observations	751		
R^2 / R^2 adjusted	0.005/0.001		

Values are fitted parameters and 95% confidence intervals (CI) from linear models. P-values < 0.05 are bolded. Number of observations and R^2 values are listed below each model.

New Phytologist

Table S6 Association of (a,b) mean leaf mass per area (LMA) (g m^{-2}) and (c,d) mean leaf nitrogen (N) content (mg g^{-1}) with resprouting and post-fire seeding (both coded as TRUE or FALSE), including woody or herbaceous growth form as an explanatory factor and any interactions and **perennial species only**.

	Estimates	*CI*	*P*
**(a) Mean LMA [log10] versus resprouting**			
(Intercept)	1.88	1.82 – 1.94	**<0.001**
Resprouting	0.12	0.06 – 0.18	**<0.001**
Woody or herb [linear]	0.39	0.32 – 0.45	**<0.001**
Resprouting * Woody or herb [linear]	-0.16	-0.23 – -0.09	**<0.001**
Observations	2750		
R^2 / R^2 adjusted	0.124/0.122		
**(b) Mean LMA [log10] versus seeding**			
(Intercept)	2.08	2.01 – 2.14	**<0.001**
Seeding	-0.13	-0.21 – -0.05	**<0.001**
Woody or herb [linear]	0.02	-0.05 – 0.09	0.578
Seeding * Woody or herb [linear]	0.28	0.19 – 0.36	**<0.001**
Observations	1461		
R^2 / R^2 adjusted	0.128/0.126		
**(c) Mean leaf N content [log10] versus resprouting**			
(Intercept)	1.23	1.17 – 1.28	**<0.001**
Resprouting	-0.05	-0.12 – 0.01	0.120
Woody or herb [linear]	-0.14	-0.21 – -0.08	**<0.001**
Resprouting * Woody or herb [linear]	0.07	0.001 – 0.14	**0.046**
Observations	1618		
R^2 / R^2 adjusted	0.025/0.024		
**(d) Mean Leaf N content [log10] versus seeding**			
(Intercept)	1.18	0.02 – 2.25	**<0.001**
Seeding	0.07	-0.17 – 0.12	0.182
Woody or herb [linear]	-0.04	-0.17 – 0.14	0.416
Seeding * Woody or herb [linear]	-0.10	-0.16 – 0.14	0.072
Observations	866		
R^2 / R^2 adjusted	0.028/0.024		

Values are fitted parameters and 95% confidence intervals (CI) from linear models. P-values < 0.05 are bolded. Number of observations and R^2 values are listed below each model.

References

Simpson KJ, Jardine EC, Archibald S, Forrestel EJ, Lehmann CER, Thomas GH, Osborne CP. **2021**. Resprouting grasses are associated with less frequent fire than seeders. *New Phytologist* **230**: 832–844.
